# Dynamic Silk Fibroin Hydrogels for Programmable Bioactuation and Smart Shape Deformation: Mechanisms, Performance Evaluation, and Biomedical Applications

**DOI:** 10.3390/gels12070654

**Published:** 2026-07-21

**Authors:** Asim Mushtaq, Khai Ly Do, Taswar Ahsan, Shoaib Ashiq, Weizhu An, Miao Su, Muhammad Yousaf

**Affiliations:** 1 College of Textile Science and Engineering (International Silk Institute), Zhejiang Sci-Tech University, Hangzhou 310018, China; asimmushtaq@zstu.edu.cn (A.M.);; 2 Shengzhou Innovation Research Institute, Zhejiang Sci-Tech University, Shengzhou, Shaoxing 312400, China; 3 Zhejiang Provincial Key Laboratory of Silk and Silk Protein New Materials, Hangzhou 310018, China; 4 Institute of Plant Protection, Liaoning Academy of Agriculture Sciences, Shenyang 110161, China; 5 Department of Chemistry, University of Gujrat, Gujrat 50700, Pakistan; 6 Future Science Research Institute, ZJU-Hangzhou Global Scientific and Technological Innovation Center, Zhejiang University, Hangzhou 311200, China

**Keywords:** silk fibroin hydrogels, programmable deformation, bioactuation, stimuli-responsive materials, 4D bioprinting

## Abstract

Programmable hydrogel actuators represent an innovative group of adaptive soft matter systems, which are able to respond to external stimuli with controllable mechanical movements for biomedical and bioengineering purposes. Natural silk fibroin (SF) is known to be a peculiar biomaterial, since it can exhibit controllable β-sheet-induced structural transitions, hierarchical self-assemblies, high biocompatibility, and mechanical adaptability, thus representing an ideal candidate for the development of dynamic hydrogels. In contrast to earlier reviews which focused more on SF hydrogel synthesis or biomedical applications, this review presents a mechanism-based understanding of programmable bioactuation by carefully correlating molecular design, network formation, stimuli responsiveness, and macroscopic deformation. Recent developments in SF hydrogel actuators are critically compared in terms of actuation principles, deformation behaviors, response dynamics, mechanical robustness, and functionalization, noting the natural compromise between fast response, strength generation, and durability in such materials. Novel concepts like nanocomposite materials, bioinspired designs, shape memory systems, and 4D printing are described as efficient ways to improve programmable deformation and functionality in soft materials. In addition, the biomedical opportunities of responsive SF hydrogels in wound healing, drug delivery, tissue engineering, wearable biosensors, and soft robots are critically discussed in relation to existing barriers for translation into practice. Combining mechanistic understanding with the comparative assessment of the performance of hydrogels is a basis for developing a complete rationale for the design of the next generation of SF hydrogel actuators and smart shape deformations.

## 1. Introduction

Hydrogels are hydrophilic polymeric networks that retain large quantities of water due to crosslinking that is physical or chemical in nature [1]. Due to their highly hydrated nature, unique physical/chemical properties, and biomechanical features, hydrogels have emerged as one of the most widely studied soft materials for biomedical and engineering applications [2,3]. The recent advent of stimulus-sensitive hydrogels has led to an important extension of the basic functions of hydrogels by endowing them with abilities for reversible deformation and adaptive mechanical behavior in response to stimuli [4,5]. Such advanced hydrogels have gained increased interest in various applications including soft robotics, artificial muscles, wearable devices, biosensing, tissue engineering, drug delivery systems, and intelligent biomedical devices [6,7].

The ideas of programmable deformation and bioactuation pertain to recent trends in soft materials of the future when hydrogels exhibit programmed movement, deformation, or mechanical behavior in an exact and reversible way. Dynamic hydrogels differ from static counterparts by the ability to change their structure and properties due to external factors like pH, temperature, irradiation by light, humidity, magnetic field, and electric current [8,9]. The development of hydrogels demonstrating these properties is of great interest because they may be used in the creation of smart materials able to imitate biological motions and functions. Bioinspired soft actuators using hydrogels have shown high prospects for the realization of various biological motions occurring in muscles, tendrils, flowers, and other organisms [10,11]. However, the combination of rapidity, durability, and multiplicity represents a major problem for existing hydrogels [12].

To overcome such drawbacks, recent emphasis is being put on developing biomolecules extracted from natural sources with inherent properties like biocompatibility and adaptable structure. Silk fibroin (SF), which can be extracted from the Bombyx mori silkworm, has gained immense attention due to its promising potential for hydrogel fabrication [13]. When compared to popular hydrogels like GelMA, alginate, and PNIPAM, SF has several unique properties, which include its remarkable mechanical strength; biodegradability with a controlled degradation rate ranging from a few days to many months; low immunogenic properties; optical clarity; and a diverse ability to be fabricated into film, fiber, sponge, nanoparticles, and hydrogels [14,15]. Most importantly, the conformational change from random coil to alpha helix and then β-sheets, which can be achieved by exposing SF to certain external stimuli, has great potential in controlling the rigidity and elasticity of the hydrogels [16,17].

The recent revolution in materials science and nanotechnology has also led to an increase in the pace of innovation regarding dynamic SF-based hydrogels [18]. Using functional nanomaterials such as graphene oxide (GO), MXene (Ti_3_C_2_T_x_), carbon nanotubes, metallic nanoparticles, and magnetic nanoparticles, SF-based hydrogels can exhibit high electrical conductivity, photothermal efficiency, electroactivity, and multiple stimulus responses [19,20,21,22,23]. These hybrid constructs have helped in developing smart actuators that are able to bend, twist, fold, contract, and even heal themselves based on specific stimuli [24,25]. Furthermore, novel fabrication techniques such as 3D and 4D printing, gradient assembly, and programmable crosslinking methods have paved the way for the development of complex SF hydrogels [26,27,28].

**Figure 1:**
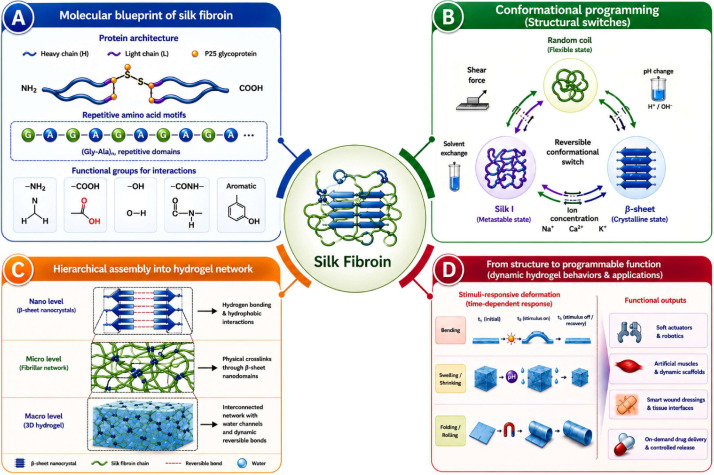
From the molecular blueprint to the programmable functions of the hydrogel using silk fibroin. (**A**) The molecular blueprint of silk fibroin, (**B**) structure-based programming of the hydrogel properties, (**C**) hierarchical assembly of the hydrogel network, and (**D**) hierarchical structure to programmable functions.

Although there have been remarkable advancements in this area, most previous review articles published concerning the topic of SF hydrogels deal more with applications, which range from general biomedical applications, drug delivery systems, tissue engineering, and mechanical reinforcement. A comprehensive critical evaluation of the research efforts undertaken concerning programming deformations, comparative performance of actuators, and intelligent bioactuations in this field is still rare [11,29,30]. It is important to understand how the structure transition of SFs is related to their stimulative response and intelligent actuation mechanism. This review highlights recent progresses of dynamically deformable silk fibroin hydrogels towards controllable bioactuation and intelligent deformation. Firstly, structural and physiochemical features of SF are reviewed and compared to other competing biomaterials. Then, important techniques of fabrication and crosslinking are presented and critically assessed considering their mechanical properties, responsiveness and scalability. Particular attention is paid to stimuli-responsive deformation mechanisms including those triggered by changes in pH, temperature, light, electricity and magnetism. Important actuation parameters such as response time, actuation strain, and reversibility are discussed in detail. New advances in shape memory materials, self-folding structures, bioinspired actuators, and 4D-printed SF hydrogels are critically assessed as presented in Figure 1.

## 2. Structural and Physicochemical Features of Silk Fibroin for Dynamic Hydrogels

### 2.1. Molecular Structure of Silk Fibroin

Silk fibroin (SF) is a natural protein fiber obtained from the cocoons of silkworms, Bombyx mori, using a standardized degumming and dissolution technique [31]. The distinct molecular structure of SF is mechanistically important in defining its properties with respect to mechanical performance, structural flexibility, and stimuli responsiveness in hydrogels, which together exceed the capabilities of other competitive single-component biomaterials [32]. Natural silk fibroin comprises a large chain (~300 kDa), small chain (~30 kDa), and glycoprotein P25 chain bound by a disulfide and hydrophobic interaction [33]. The large chain has repeating hydrophobic motifs that lead to a β-sheet structure, whereas hydrophilic domains mediate solubility, elasticity, and molecular stability [34].

Among the most important features in SF that contributes to its superiority in terms of designability for dynamic hydrogels is the capability of SF molecules to switch between random coil, Silk I (metastable α-helical), and β-sheet conformations depending on the environmental factors. It should be noted that β-sheet regions constitute physical crosslink sites that confer mechanical strength and stiffness; in contrast to chemical crosslinks, they can be easily modified. Indeed, the proportion of β-sheets can vary from ~10% in solution to >50% in stiff gels, leading to moduli of elasticity that range from ~0.5 kPa to >300 kPa without changing the chemical structure [35,36]. Such an incredible variability in the mechanical properties of SF has no match in GelMA or alginate-based materials [37,38,39]. β-sheet formation is inducible by various stimuli including temperature, pH, shear stress, sonication, organic solvents, salt content, and light, thus enabling multi-trigger conformation switching [32,40].

Chemically, SF is highly reactive due to the presence of various functional groups in it (amino (–NH_2_), carboxyl (–COOH), hydroxyl (–OH), and aromatic), thus opening many routes for conjugations and hybridizations [41]. This feature is one of the biggest advantages that SF possesses compared to alginate, which can only undergo modification via carboxyl groups [42]. Due to the reactivity of functional groups in SF, the material may be chemically bonded with different nanoparticles, conductive materials, bioactive substances, and artificial polymers to obtain multifunctional hydrogels [43,44]. Another great advantage of SF is its high optical transparency and tunable degradation properties (from days to months based on crystallinity) [45].

### 2.2. Key Physicochemical Properties Supporting Dynamic Behavior

The physiochemical nature of SF makes it a unique material that is able to act as an excellent candidate for developing dynamic hydrogels. Table 1 offers a comparison between SF and other important hydrogels, such as GelMA, alginate, and PNIPAM hydrogels.

As demonstrated in Table 1, SF stands out as being uniquely capable of delivering wide-range mechanical tunability (0.1–500 kPa), multi-stimuli responsiveness due to intrinsic conformational transitions, FDA-compliant biocompatibility, and adjustable biodegradability in one natural biomaterial system [50,51,52]. Although GelMA displays improved cell-adhesion properties and is amenable to fabrication by bioprinting, its multi-stimuli responsiveness cannot be achieved unless supplemented with a hybrid synthetic approach [53,54]. Alginate demonstrates high scalability and safety but exhibits limited responsiveness to ionic and acidic environments alongside weak mechanical stability [55,56]. Thermo-responsive hydrogels formed by PNIPAM show rapid and reversible phase transition at about 32 °C; however, they are non-biodegradable/nondegradable unless chemically modified, and cytotoxicity due to left-over monomers may become an issue [57,58]. Therefore, SF finds itself at a unique advantage especially for multi-stimuli actuator functions that cannot be duplicated by any wholly artificial or natural mono-functionalized biomaterials.

**Figure 2:**
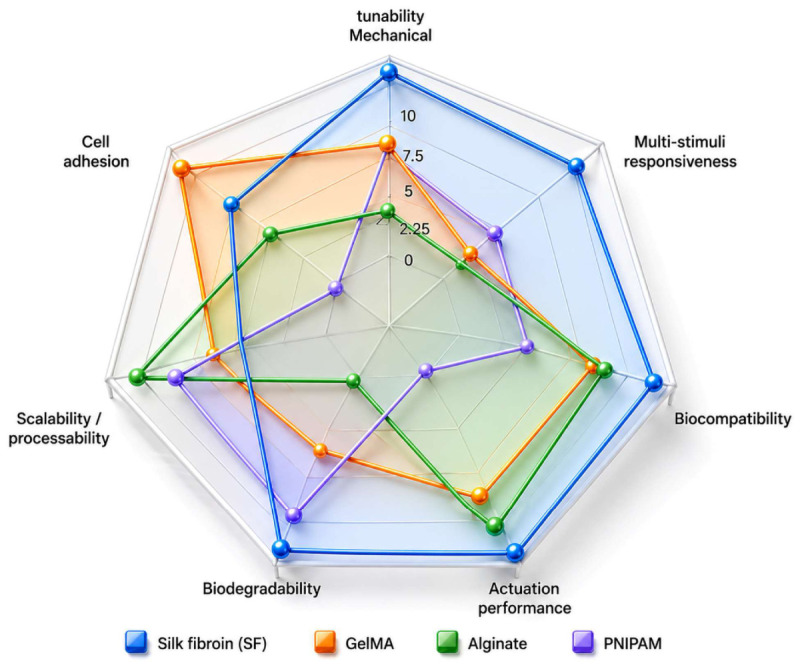
Spider graph showing the comparison of the silk fibroin (SF) hydrogel against the other three materials (GelMA, alginate, and PNIPAM hydrogels) based on the following seven features which are important for the development of programmable actuators. The silk fibroin exhibits the broadest range of properties and is superior in these aspects mentioned in Table 1. Scores are derived from comparative data summarized in Table 1.

The tunability of SF hydrogel swelling and deswelling is one of its most interesting features and directly relates to the deformation capacity of these materials. The absorption of water leads to the expansion of hydrogel material in volume while dehydration results in contraction; thus, swelling levels of 200–800% can be achieved through appropriate adjustment of crosslinking density and the proportion of β-sheet structure formation [59,60]. Additionally, through regulation of the mentioned characteristics, the swelling rate and behavior of SF hydrogels can be regulated [61]. Another important biocompatibility factor is SF’s FDA approval and low inflammatory response to SF along with its high cytocompatibility and ability to degrade into nontoxic amino acids [62,63]. The features listed above make SF a highly attractive biomaterial for designing advanced hydrogels and make its rating high as presented in Figure 2.

## 3. Fabrication Strategies of Dynamic Silk Fibroin Hydrogels

The method used for the fabrication of SF dynamic hydrogels influences their mechanical properties, actuation ability, and responsiveness. Most importantly, none of these methods can fulfill all the requirements at once; physical crosslinking is suitable because of its reversibility and biocompatibility; chemical crosslinking ensures high strength but may pose cytocompatibility challenges; nanocomposite hybridization allows for multifunctionality yet raises safety issues. It is important to understand these limitations when designing SF hydrogels, as described in Table 2.

### 3.1. Physical Crosslinking Strategies

**Figure 3:**
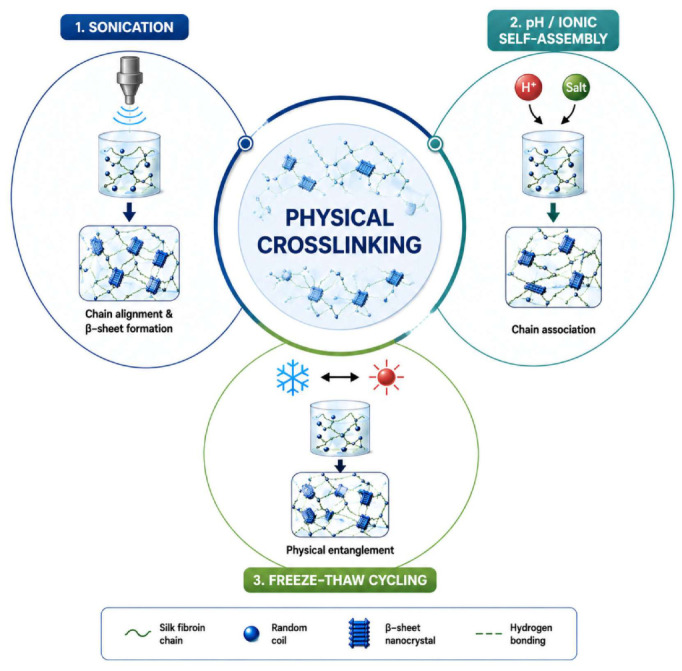
Physical crosslinking approaches include: (1) gelation through sonication which causes β-sheet formation and alignment of polymer chains (minutes-long process, no need for additives); (2) gelation through pH/ionic induction which causes interchain interactions due to changes in the surrounding environment; (3) gelation via freeze–thaw cycles which results in ice-templating of porous materials and physical entanglement of polymer chains.

The physical crosslinking method can be considered one of the most common techniques for the preparation of SF hydrogels (Figure 3). This technique is characterized by the simplicity of the process and mild synthesis conditions and is free from toxic crosslinkers [39,64]. The main principle of forming physically crosslinked hydrogels is the participation of non-covalent interactions, such as hydrogen bonding, hydrophobic interactions, and β-sheets self-assembly [65]. Of all the techniques for forming hydrogels, sonication gelation is applied more commonly; in this approach, the gelation of SF solution takes place under the influence of ultrasonication (20–60 W for 1–30 min), which promotes the formation of β-sheets; the formation of a hydrogel with a storage modulus (G’) ranging from 100 to 10,000 Pa is observed [66,67]. One of the major benefits of the technique is the absence of any additional substances in SF hydrogels, which renders it useful in clinical applications [65].

Gelation through self-assembly occurs under pH (4–6) or ionic strength (>0.1 M NaCl), resulting in hydrogels having a G′ generally less than 1 kPa—less mechanically durable but more physiological as compared to sonication-induced or chemical gels [18,68]. Self-assembled hydrogels possess full structural flexibility that enables stimuli-responsive deformations, but their lack of mechanical endurance makes them inadequate for cyclic loading systems. Hydrogel fabrication through freeze–thaw methods leads to SF hydrogels being porous with a pore diameter ranging from 50 to 300 µm with an improved capacity for water retention [69]. The significant increase in porosity is ideal for tissue engineering applications; however, the method’s limitations are time-consuming (>3 cycles in 24–48 h) and there is a risk of protein denaturation due to extreme temperature.

### 3.2. Chemical and Enzymatic Crosslinking Strategies

**Figure 4:**
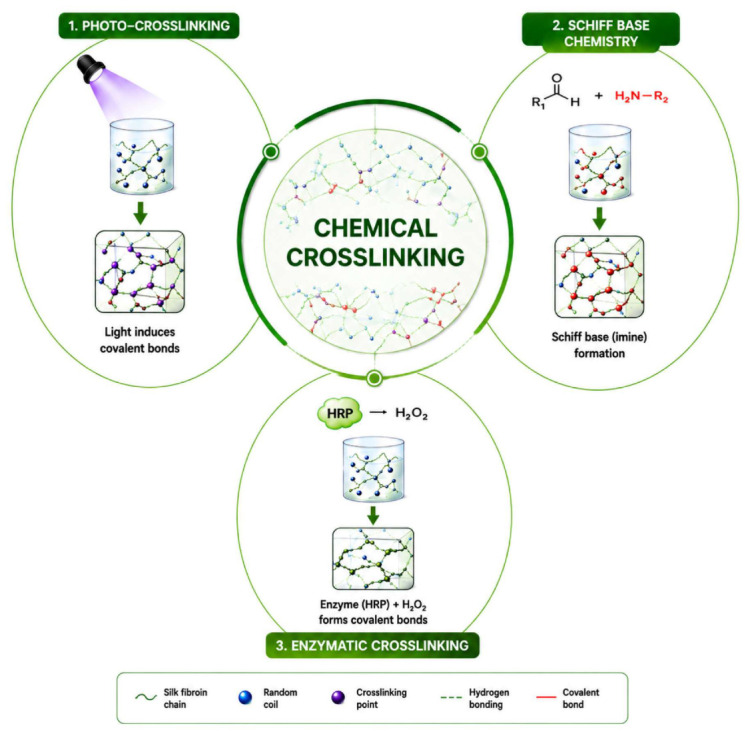
Chemical crosslinking includes; (1) photo-induced crosslinking of methacrylate-functionalized SF (SilMA) in response to ultraviolet/visible light leading to controllable gelation; (2) formation of reversible imine linkages (–C=N–) through Schiff base reaction which facilitates self-healing properties and injection; (3) enzymatic crosslinking using horseradish peroxidase/hydrogen peroxide and tyrosine residue coupling to form biocompatible covalent networks.

Chemical crosslinking (Figure 4) allows for the creation of hydrogel network systems that possess improved mechanical properties and controlled degradation in comparison with physical gelation approaches [70,71]. Methacrylate-modified SF gelation (SilMA) using photo-crosslinking under UV or visible light and photo-initiators has been recognized as the best approach for precise SF hydrogel manufacturing, allowing for the tuning of G′ from 1 to over 100 kPa through light dosage and SilMA concentration [72,73]. The key benefit over physical gelation lies in the spatial and temporal control of gelation; it takes just a few seconds for gelation to occur in a targeted location and hence it is perfectly suitable for 3D/4D bioprinting applications [74]. The main drawback here is related to the cytotoxicity of photo-initiators used at high UV levels, which should be carefully controlled in order to achieve over 85% of cell survival rates [72]. Unlike GelMA photo-crosslinking, SilMA hydrogels exhibit better mechanical adaptability and multi-stimuli responses via the formation of β-sheets [71].

Schiff base reactions generate injectable SF hydrogels, which exhibit self-healing characteristics due to reversible imine bonds (–C=N–) between aldehyde and amine groups [75,76]. In contrast to traditional crosslinks, which are covalent and permanent, reversible crosslinks are advantageous for programmable deformation devices since they have self-repair capability (>90% recovery in minutes) and stress-relaxation properties, both of which play an important role when repeating deformation cycles many times [13]. However, the relatively low strength of imine bonds (moderate G′ of 100–1000 Pa) and pH sensitivity constrain their usage only to cases when actuation cycles include mild deformations instead of high-force generation. Hydrogels based on enzymatic crosslinking by horseradish peroxidase/H_2_O_2_-induced dityrosine bonds provide gels with adjustable strength G′ values reaching up to 50–5000 Pa at fully physiological conditions (37 °C, pH 7.4) [63,77,78]. This method exceeds chemical crosslinking in cytocompatibility while being inferior in gelation speed compared to photo-crosslinking.

In comparison, physical and chemical crosslinking techniques constitute two different design approaches that can be utilized in creating SF hydrogels. While the physical approach maintains the biological activity of SF while allowing dynamic deformations of the material via non-covalent interactions, the instability and the nature of the interactions lead to a lack of strength and stability of the resultant materials. In turn, the chemical crosslinking technique creates robust and stable structures, and it offers precise control over the mechanical characteristics; however, it can potentially decrease the dynamic capability of the network because of the formation of irreversible covalent bonds. Thus, neither strategy is universally superior as both of them are characterized by different advantages. Physical or reversible crosslinking techniques should be used for creating injectable, self-healing, and actuatable systems, while chemical techniques should be considered in cases when mechanical robustness and stability of the hydrogel are needed. Novel strategies for hybrid crosslinking that involve physical crosslinks together with chemically stabilized networks could be a way to design SF hydrogels with improved strength, responsiveness, and functionality.

### 3.3. Hybrid and Nanocomposite Silk Fibroin Hydrogels

**Figure 5:**
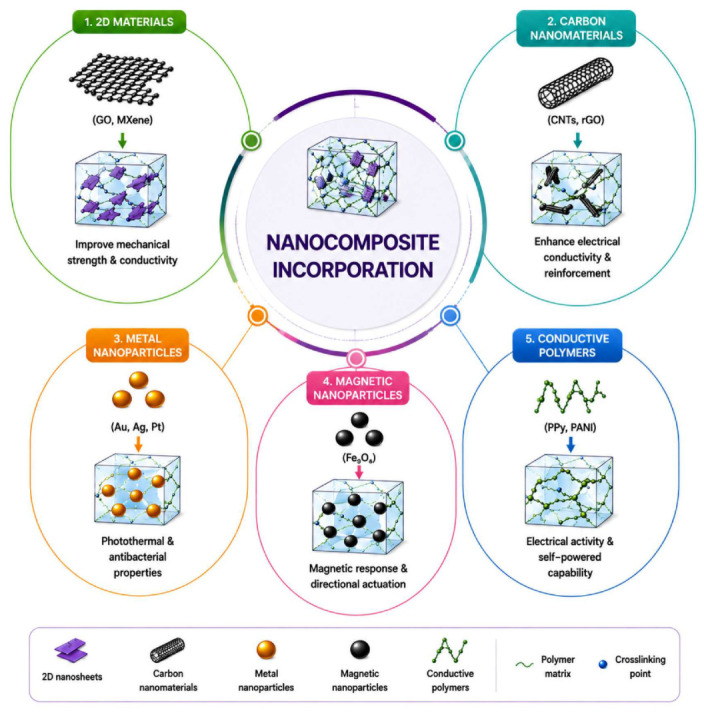
Approaches towards incorporating nanomaterials based on their types: (1) 2D materials (e.g., GO, MXene) for photothermal and conductivity effects; (2) carbon-based materials (e.g., CNTs, rGO) for mechanical/electrical performance enhancement; (3) metal nanoparticles (e.g., Au, Ag, Pt) for photothermal/antibiotic effects; (4) use of magnetic nanoparticles (Fe_3_O_4_) for magnetic activation; (5) conductive polymers (PPy and PANI) for biodegradable electroactivity.

The incorporation of nanomaterials (Figure 5) into SF hydrogels aims to overcome the drawback of insufficient functionality for complex actuation by incorporating properties such as conductivity, photothermal behavior, magnetism, and mechanical strength [79]. It is noteworthy that the choice of the nanomaterial affects the desired properties achieved and the potential biosafety issues. Between the 2D nanomaterials, MXenes (Ti_3_C_2_T_x_) exhibit higher photothermal conversion efficiency (more than 40% compared to graphene oxide (GO) with approximately 20–30%) and a higher range of absorption of NIR radiation, together with better electrical conductivity (up to 10^4^ S/m) [80,81,82,83]. MXene-based SF hydrogels are thus more suitable for applications involving NIR-induced actuation and bioelectronics [84,85]. Nevertheless, the stability and the degradation products of MXenes need to be determined in more detail. On the contrary, the surface chemistry of GO is better suited for chemical modifications, as well as having proven biocompatibility [83,86].

Carbon nanotubes (CNTs) improve electrical conductivity as well as mechanical strength, but due to their known cytotoxicity even in high concentrations, they have less appeal compared to MXenes for biomedical actuators except for surface-functionalized forms [85,87,88]. Metallic nanomaterials, on the other hand, exhibit high levels of specificity: gold nanorods possess the highest photothermal sensitivity in the NIR window (800–900 nm), whereas Fe_3_O_4_ nanomaterials (5–20 nm) facilitate remote magnetic control based on directed force generation or magnetothermal heating (mN level) [89,90]. Notably, the combination of Fe_3_O_4_ and photothermal nanomaterials yields dual-trigger SF hydrogels, wherein magnetic and NIR controls could be employed independently [23], apart from signal interference described later in Section 7. The use of conductive organic polymers (polypyrrole, polyaniline) allows for an entirely biodegradable alternative to inorganic nanofillers, although lower conductivity limits their performance as electroactive SF hydrogels (<0.1–10 S/m) [91,92]. These methods can be seen in brief forms in Table 2 below.

**Table 2:** Methods used to make silk fibroin hydrogels and their influence on the characteristics of silk fibroin hydrogels. (G′: storage modulus; HRP: horseradish peroxidase; MXene: Ti_3_C_2_T_x_; GO: graphene oxide; CNTs: carbon nanotubes).

Fabrication Method	Mechanism	Key Features	Advantages	Limitations	Typical Applications	Ref.
Sonication-induced gelation	Ultrasound promotes β-sheet formation via chain rearrangement; gelation time of 5–30 min depending on power	Rapid gelation, tunable porosity (50–200 µm)	Simple, solvent-free, injectable	Limited long-term stability; power inconsistency affects batch reproducibility	Injectable hydrogels, wound dressings	[66,93]
Self-assembly (pH/ionic)	Environmental triggers (pH 4–6 or ionic strength > 0.1 M) drive H-bonding and hydrophobic association	Mild conditions, reversible networks	Biocompatible, no additives required	Weak mechanical strength (G′ < 1 kPa typical); slow gelation	Drug delivery, soft scaffolds	[18,68]
Freeze–thaw cycling	Repeated freeze (−20 °C)/thaw cycles induce phase separation and physical entanglement	Porous structure, improved water retention	Enhanced flexibility and porosity; no chemical agents	Time-consuming (≥3 cycles); risk of protein denaturation	Tissue engineering scaffolds	[69]
Photo-crosslinking	Photo-crosslinked silk fibroin; crosslinking density tunable by light dose	Spatiotemporal gelation control; G′ 1–100 kPa	High precision, 3D/4D printing compatible	Requires photo-initiators; potential cytotoxicity at high UV dose	Bio-fabrication, patterned hydrogels	[94,95,96]
Schiff base chemistry	Dynamic imine bonds (–C=N–) between aldehyde and amine groups; reversible under pH/temperature change	Self-healing (recovery > 90%), injectable	Adaptable structures; self-repair under cyclic loading	Moderate mechanical strength; stability sensitive to pH	Self-healing actuators, drug delivery	[75,76]
Enzymatic crosslinking (HRP/H_2_O_2_)	Tyrosine oxidation by HRP forms covalent dityrosine bonds; gelation at 37 °C within minutes	Physiological conditions; tunable G′ 50–5000 Pa	Excellent biocompatibility; precise mechanical control	Slower gelation; H_2_O_2_ concentration must be optimized	Tissue engineering, cartilage repair, implants	[78,97]
Nanocomposite incorporation	Integration of MXene, GO, CNTs, Fe_3_O_4_ into SF matrix via physical blending or covalent grafting	Multi-functionality; conductivity up to 10 S/m; photothermal efficiency > 40%	Multi-stimuli actuation; enhanced mechanical and electrical properties	Potential nanotoxicity; dispersion uniformity challenging	Soft robotics, bioelectronics, photothermal therapy	[23,85,87]

## 4. Stimuli-Responsive Silk Fibroin Hydrogels for Smart Deformation

**Figure 6:**
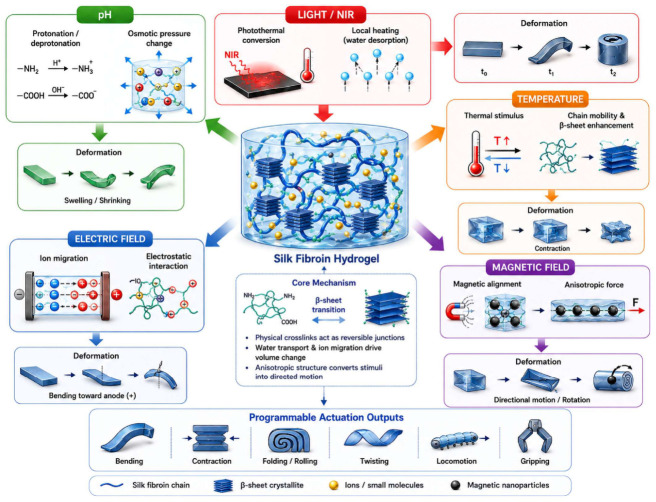
A schematic illustration of multi-stimuli-responsive deformation mechanisms in silk fibroin hydrogels.

Hydrogels that exhibit stimuli responsiveness can undergo reversible physical and mechanical changes induced by an external stimulus, which allows for programmable deformation and intelligent actuation [65,98]. Conformational changes, swell/deswell, and structure alterations in SF-based hydrogels rely on the reversible switching between β-sheets mechanism mentioned above in Section 2 [99]. A critical comparative analysis approach is necessary: while every type of stimulus has its unique features, it can significantly differ from another in terms of reaction time, spatial resolution, depth of tissue penetration, and suitability for particular applications. Choosing an adequate stimulus goes beyond materials science into systems engineering territory. Figure 6 presents the multi-stimuli-responsive deformation mechanism of SF-hydrogels.

### 4.1. pH-Responsive Silk Fibroin Hydrogels

pH-sensitive SF hydrogels rely on the ionization of amino groups (pKa ≈ 8) and carboxyl groups (pKa ≈ 3.5) to alter swelling via electrostatic repulsion and osmotic pressure changes [100,101]. Swelling ratios between 200 and 800 percent can be achieved over a pH range of 4 to 10 with reversibility on the minute to hour timescale dependent on the hydrogel thickness; it is slower than other methods such as light and magnetic actuation but it is intrinsically biologically relevant [65,100]. This significant improvement over other stimuli lies in the natural pH fluctuations in physiological conditions—infected tissues (pH 5–6), cancer cells (pH 6–6.8), and gastrointestinal tracts (pH 1–8) [101]. Combining SF with other bio-polymers such as chitosan, alginate, and poly(acrylic acid) improves the sensitivity while retaining the biocompatible nature, resulting in actuators or drug delivery systems with direct application [102,103]. Differential swelling caused by pH gradient leads to directional actuation with curvature as high as 0.5 cm^−1^, useful for gripper applications and actuators [104,105]. One significant disadvantage compared to light and magnetic actuation is the diffusion-dependent delay and inability to control the system on/off remotely within the body.

### 4.2. Thermo-Responsive Silk Fibroin Hydrogels

Although temperature-responsive SF hydrogels respond fast and reversibly, they need either the additional use of heating devices or the incorporation of photothermal nanomaterials to provide controllable actuation. Pure SF hydrogels exhibit a reversible temperature-induced transition from α-helical to β-sheet crystallization (β-sheet formation increases when the temperature is increased from 40 to 60 °C), which results in a network stiffening; however, they do not demonstrate a sharp lower critical solution temperature (LCST) transition by themselves [59,106,107]. Upon hybridization with PNIPAM, SF hydrogels demonstrate a sharp volume transition at LCST (≈32 °C), allowing reversible volume changes of up to 80% [108,109]. Notably, hybrid systems based on SF and PNIPAM are superior to those with pure PNIPAM in terms of biocompatibility and robustness; however, the response time to stimuli in them is not as fast as in the case of MXene-SF and AuNR-SF photothermal systems. An especially important clinical feature of PNIPAM-SF systems is that they allow changes in the LCST from 25 to 40 °C via varying copolymer composition, enabling body temperature-activated actuation without applying any external stimuli to the system [20,110]. The combination of photothermal effects using MXenes or gold nanorods transforms the near-infrared radiation into heat energy with a temperature difference (ΔT) of up to 30 °C in seconds, which significantly increases the speed compared to the conventional thermal bath methods and enables remote activation [110,111]. This is the principle behind the fastest known SF actuators.

### 4.3. Light-Responsive Silk Fibroin Hydrogels

Photo-responsive SF hydrogels offer the highest spatial and temporal precision among actuation mechanisms, which make it possible to reach sub-millimeter resolutions of deformation in a remote and non-invasive manner [40,112]. Such a high level of precision compared to pH or magnetic stimulation is attributed to the possibility of focusing, patterning or masking the beam of light at the micron scale [113,114]. Near-infrared light, with wavelengths ranging between 700 and 1000 nm, has been used widely in biomedical engineering owing to the relative transparency of biological tissue in the NIR window, making it possible to perform actuations several millimeters deep into tissues [115,116]. As far as photothermal properties are concerned, MXene–SF hydrogels have shown the best results amongst all the SF-based actuators, thanks to Ti_3_C_2_T_x_ MXene which exhibits photothermal conversion efficiencies over 40% and produces temperature rises of up to 20–30 °C within 5–10 s of 808 nm irradiation at 1–2 W/cm^2^ [82,111]. Although GO-SF systems have demonstrated relatively poor photothermal efficiency (~20–30%), they exhibit improved chemical functionalization capabilities, in addition to proven biocompatibility characteristics, rendering them more favorable for cell-laden actuators [19]. The main drawback of light-actuated SF systems is the limited depth to which NIR light penetrates biological tissues (up to ≤5 mm).

### 4.4. Electro-Responsive Silk Fibroin Hydrogels

Electro-active SF hydrogels represent the overlap between the fields of soft actuators and bioelectronics due to their ability to use electrical impulses not only to generate a movement but also to produce a signal [117,118]. Such bidirectional behavior, impossible with respect to pH, thermal, or magnetic systems, allows the use of electro-active SF hydrogels for closed-loop applications [119]. Pure SF hydrogel shows very low electrical conductivity (10^−10^ S/m), thus necessitating the inclusion of nanofillers [118,119]. Conductivity levels achieved with various combinations of MXene–SF, CNT–SF, and polypyrrole–SF are sufficient for electro-actuation with voltages of 1–10 V (0.1–10 S/m) [117,120,121]. The advancement in electro-active SF hydrogels showed the possibility of creating gradients within the network of SF hydrogels by electric field manipulation of chain movements, generating negatively charged gradient networks capable of reconfiguring their shape (2D to 3D) in a variety of ways—a feat never before seen in any other material, including GelMA or alginate [117,119]. Electro-actuation represents one of the fastest stimulus types discussed here in terms of response (sub-second) and voltage required (compatible with existing bioelectronics hardware) [122]. The main limitation associated with electro-actuators is the need for wiring/electrodes.

### 4.5. Magnetic-Responsive Silk Fibroin Hydrogels

In terms of untethered actuation methods, magnetic-responsive SF hydrogels present the dual advantage of remote wireless actuation and infinite tissue depth penetration theoretically feasible for magnetic fields. Fe_3_O_4_ nanoparticles of sizes ranging from 5 to 20 nm within SF matrix concentrations of 0.5–5 wt % have been found to be sufficient in creating directional magnetic actuation forces without impairing material flexibility [123,124]. Magnetic fields (10–100 mT) have created enough alignment-induced magnetic forces to initiate motion, rotation, bending, and directional movement in narrow spaces and that is a process observed for SF-based soft microrobots moving in fluid-filled channels [123,125]. More importantly, magnetic actuation is the sole method capable of navigating a hydrogel system through biological tissues without direct physical contact, thus enabling new avenues for targeted drug delivery and minimally invasive microsurgical operations. 4D-printed magnetic SF–gelatin constructs have proven the ability of magnetic actuation to guide tissue-level mechanical stimulation and chondrogenic differentiation simultaneously. Although magnetic actuation responds faster than any other external stimuli, its current clinical application is limited due to the need for external bulky electromagnet apparatuses [126,127].

## 5. Programmable Bioactuation and Shape-Morphing Systems

**Figure 7:**
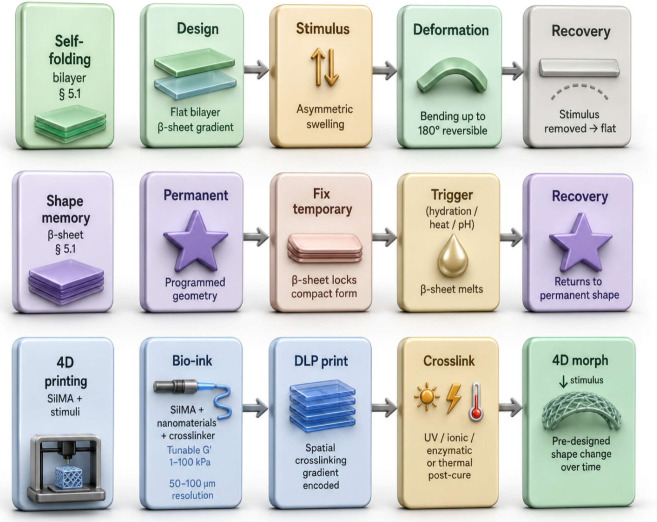
Three principal programmable actuation and shape-morphing pathways in dynamic SF hydrogels.

In addition to individual stimulus-induced reactions, the most advanced SF hydrogels utilize the structural design approach, including gradient structures, bilayers, anisotropic alignment of fibers, and digital manufacturing technologies, in order to obtain programmable bioactuation, where the pathways of deformation, motion orientation, and even temporal sequences of deformation are programmed at the material level [128,129,130]. The following section provides a critical discussion on the four main approaches to programmable SF hydrogels, including self-folding/shape memory systems, bioinspired actuators, 4D printing systems, and multi-stimuli platforms. A three principal actuation overview is given in Figure 7.

### 5.1. Self-Folding and Shape Memory Silk Fibroin Hydrogels

Self-folding SF hydrogels make use of the asymmetry in internal stresses resulting from uneven swelling or contraction in a gradient or bilayer structure to give rise to bending, curling, or folding in response to no external mechanical restriction [131]. The degree and orientation of such folding can be controlled by the distribution of β-sheet formation, crosslinking density, and swelling anisotropy within the material, all of which can be controlled in the process of fabrication [132]. Unlike the usual shape memory polymers, whose switching mechanism is usually fixed by the network structure formed during fabrication, reversible conformation changes from the random coil to β-sheet structures can be induced by stimuli like hydration, heating, and chemical agents in SF-based shape memory materials [112,133]. Environment-adaptive and adjustable recovery kinetics in the SF-controlled semi-interpenetrating network (semi-IPN) hydrogels have been recently developed to achieve hydration-induced shape recovery of hydrogels without the need for any external stimuli [134]. The use of shape memory SF hydrogels is particularly desirable in biomedical applications, where a small construct can be implanted in situ and self-deployed within the physiological fluid [135,136].

### 5.2. Bioinspired Soft Actuators Based on Silk Fibroin

Bio-organisms generate highly advanced movements utilizing the hierarchical anisotropic nature of biological tissue, such as muscle fibers, tendon-like connections, and plant cells [137,138]. SF hydrogels are highly compatible with the development of such biomimetic actuation because of their ability to undergo fibrillation, β-sheet crystallization, and integration with nanocomposites, which enable anisotropy construction at different scales [139,140]. Bioinspired SF actuator applications presented thus far include anisotropic tendon-like scaffolds with directed mechanical responses, hydration-sensitive silk systems with morphing ability, and electrically controlled silk protein-based hydrogels able to perform multimode 2D-3D morphing [141,142]. The main feature that allows high-performance bioinspired SF actuators to differ from conventional responsive hydrogels is the structural anisotropy due to which the preferred axis of deformation is defined through the directional alignment of SF chains or nanoparticles [143,144]. An example of such an application can be seen in the work of Wang et al., where the gradient structure was created with electric-field-induced protein network migration enabling programmable multimode reconfigurable shape change with deformable mode selection based on the field direction [141].

### 5.3. 4D-Printed Silk Fibroin Hydrogels

**Figure 8:**
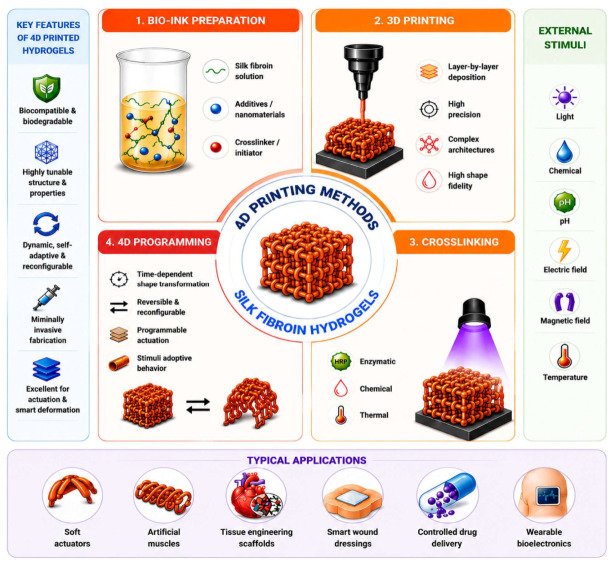
4D printing methods of silk fibroin hydrogels. The whole printing process consists of four steps including bio-ink preparation, 3D printing, and further physical and/or chemical crosslinking. Due to various stimuli-responsive effects, the printed hydrogels are able to change their shape in reaction to external stimuli like temperature, light, pH, electric field, and magnetic field. These specific properties make it possible to use them in many areas from soft actuators and artificial muscles to wound-healing bandages and even in tissue engineering and bioelectronic devices.

Bioprinting in 4D (Figure 8), which involves the construction of three-dimensional structures which exhibit programmed time-dependent morphological changes, has gained recognition as a technique for imparting complicated actuation behaviors on silk fibroin-based hydrogels [129,145].

The fourth dimension is controlled in 4D printing through material composition variation, crosslinking or swelling gradient, anisotropy of the design and layering of responsive and non-responsive sections [146]. Kim et al. developed an initial 4D bioprinting system based on photo-crosslinkable SilMA hydrogel and digital light processing (DLP) technology, where the bilayer structure exhibited predictable shape changes due to differential swelling [129,147]. The technique was successfully utilized to repair the trachea in vivo. In a follow-up silk-based minimally invasive scaffold study, swelling stiffening and shape programming was utilized in support of implantation and functional performance post-deployment, but it is different from the previous trachea work [146]. The contribution of Chakraborty et al. towards this area includes the development of 4D-bioprinted silk fibroin–gelatin magnetic structures for cartilage regeneration and how the use of magnetism improves chondrogenic performance and the link between printing process design and biological performance [127,148]. In the current situation, 4D-printed systems based on SF mostly focus on single step folding or bending of the structure [145,146]. More complex sequential movement with three or more degrees of freedom is yet to be achieved.

### 5.4. Multi-Stimuli Integrated Actuation Systems

Multi-stimuli integration is another direction of programmability of SF hydrogels, where two or even more types of stimuli are integrated into the same platform in order to perform sequence actuation, logic operations or independent actuations [40,149]. In some cases, combining different stimulus responses could generate some behaviors that cannot be achieved using only one type of stimulus [150,151]. For instance, water-responsive super contractible films based on silks have been used in rapidly adapting bioelectronic interfaces, while magnetic, electric and mechano-responsiveness were shown in related composite materials [32,152]. pH-responsive SF fibroin hydrogels and other multi-stimuli-responsive SF systems provide opportunities for stimulus-gated drug release based on multiple microenvironmental signals [32,100,153]. One of the most common problems associated with the multi-stimuli systems is the orthogonality between stimulus responses due to the fact that photothermal stimuli can activate the thermal stimulus channel, and an electrical stimulus can modify ion concentration which will act as a chemical stimulus; finding orthogonal stimulus channels in the same SF platform is still unsolved [149,150]. Recent examples such as water-responsive super-contractible silk-inspired films, multi-stimuli-responsive SF hydrogels and MXene-based multi-responsive composites suggest the development of smart soft systems capable of context-dependent actuation [152,154]. Table 3 shows the comparative data for SF-based hydrogels and their usage.

## 6. Biomedical Applications of Dynamic Silk Fibroin Hydrogel Actuators

**Figure 9:**
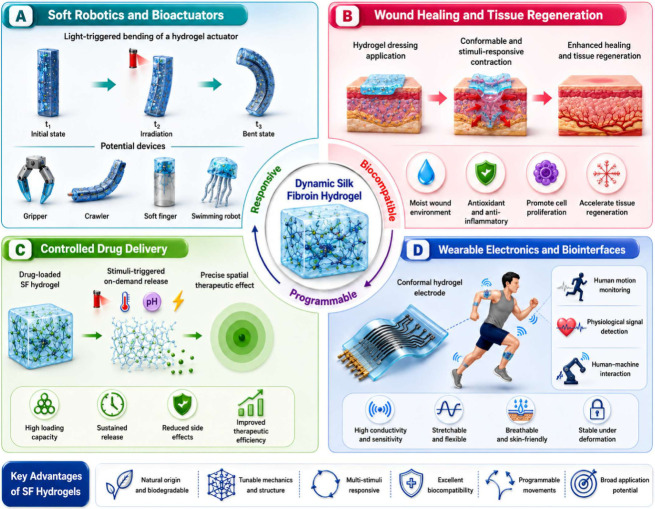
Programmable bioactuation and biomedical applications of stimuli-responsive silk fibroin hydrogels. (**A**) Soft robotics and bioactuators: Infrared-triggered curvature change in SF hydrogel actuators allows various actuation modes such as gripper, crawler, soft finger, and swimmer robot. (**B**) Wound healing and tissue engineering: Stimuli-responsive hydrogels assist in wound closure, create a moist environment, regulate inflammation, and boost cell growth and ECM regeneration. (**C**) Controlled drug delivery: Stimuli-responsive SF hydrogels allow programmable drug delivery with high drug-loading capability, controllable release kinetics, low systemic toxicity, and better therapeutic efficiency. (**D**) Wearable electronics and biointerfaces: Conductive and flexible SF hydrogels allow real-time monitoring and sensing of physiological activities as well as human–machine interactions. The bottom figure indicates the natural properties that render SF hydrogels suitable programmable biomaterials for advanced biomedical applications.

It is the unique integration of programmable deformation, biocompatibility, biodegradability, and multiple stimuli reactivity that gives SF hydrogel actuators the edge in becoming the leading biomedical innovation of the future. More importantly, SF hydrogel actuators not only assist in biological functions but they are directly involved in biological interactions both mechanically and chemically, setting them apart from typical passive biomaterials. In the next sections, the performance of SF hydrogel actuators in five key areas of application is assessed critically based on where they surpass current methods and where limitations still lie. These applications are briefly outlined in Figure 9.

### 6.1. Soft Biomedical Robotics and Minimally Invasive Devices

Dynamic SF hydrogels are being explored for use in soft biomedical robotics since their programmed deformability, biocompatibility, and multiple stimulation responses can address the mechanical mismatch issue between stiff robots and soft tissues, which leads to potential injury and inflammation [17,40]. Examples of SF-based soft robots published in the literature include light-driven robotic grippers and shape-morphing systems operated via the NIR photothermal effect [129,166]. Magnetically operated SF and SF-based microrobots have been showcased for precise navigation within narrow channels and therapeutic drug release on-demand in targeted locations [148,167]. Crawler-type robotic systems operated using a periodic magnetic field to generate displacements in the millimeter scale have been demonstrated in the context of soft robotics (typically with speeds in the 1–5 mm min^−1^ range). Also described in the larger body of soft robotics literature are soft swimming robots based on the design of jellyfish or flagella which make use of oscillatory optical or magnetic stimuli [145,168]. For instance, studies showed a bioinspired SF-based actuator that integrates actuation with self-sensing by achieving significant bending under NIR light irradiation and simultaneous production of electrical signals corresponding to the actuation, thus allowing for closed-loop control without external sensors [141,144]. One recent review on continuum soft robots emphasized that biological SF is inherently less force-dense and slower to actuate than synthetic elastomers but has notable biocompatibility and biodegradability properties that give it a leg up in in vivo applications [40]. A critical translational hurdle lies in autonomous navigation since current magnetically guided SF microbots are mostly dependent on external imaging (MRI or fluoroscopy) for navigation purposes, thus limiting their outpatient viability [141,169].

### 6.2. Smart Wound Dressings and Tissue-Responsive Interfaces

Dynamic SF hydrogels offer some of the most clinically viable applications due to their inherent sensitivity to the presence of stimuli in the wound environment such as pH changes, temperature variations, and enzymatic activity. Traditional dressings like gauzes, foams, and hydrocolloids operate on a passive principle, but SF-based dressings can have additional features like antibacterial properties, drug delivery, and tissue regeneration [17,170]. Among their reported functionalities are NIR-controlled antibacterial effects in MXene-SF composites, pH-sensitive antibiotic release in infected wounds, and contraction of the wound or shape adaptation [20,111]. In recent years, Chen et al. have shown that SF/PVB composite dressings are able to promote acute wound healing via immune modulation and extracellular matrix remodeling by decreasing the pro-inflammatory polarization of macrophages and increasing the remodeling of collagen in vivo [171]. Similarly, the functional SF hydrogel dressings with anti-oxidative and anti-bacterial properties are able to decrease healing time in diabetic wound models [170,172]. Even though there have been significant advances in SF composite technologies, the lack of comparison studies with the existing dressings in human clinical trials remains one of the main limitations of their translation into practice.

### 6.3. Tissue Engineering and Regenerative Medicine

SF-based hydrogels for tissue engineering applications do not simply act as scaffold structures but serve as mechanical microenvironments for cells capable of actively guiding cellular behavior. Mechanical stimulation is nowadays known as one of the regulators of stem cell development: compression induces chondrogenic differentiation, tension induces myogenic differentiation, and changing substrate stiffness or compressional strains affects fibroblast–myofibroblast transition [173,174]. These types of stimulations can be mimicked by SF-based dynamic actuating hydrogels; thus, simple scaffolds can turn into microenvironmental simulators [173,175]. As demonstrated by Zhao et al., the protein conformational changes within the microenvironment in SF hydrogels, particularly the transition from random coil to β-sheet structures during the gelling process, affect the behavior of the stem cells encapsulated within them and lead to the formation of cartilage-like matrices (collagen II and aggrecan) in 3D culture in the absence of exogenous growth factors [60]. SF-based hydrogels have been used for the development of cartilage organoids and osteoarthritis models, and it was found that the mechanical properties of SF more accurately reflect the dynamics of the extracellular matrix of native cartilage compared to several other synthetic hydrogels [175,176]. Importantly, the rate of degradation of the scaffold should be matched with the rate of tissue infiltration, and SF can be engineered to degrade at different rates (controlled by β-sheet crystal formation) [177].

### 6.4. Controlled Drug Delivery and On-Demand Therapeutics

The dynamic nature of SF hydrogels provides an entirely novel delivery platform where instead of passive diffusion alone, drug delivery may be controlled, sustained, or triggered by environmental factors based on changes in swelling, degradation, network configuration, and nanoparticle arrangement [178,179]. As such, this would provide far greater control over drug dosing compared to passive diffusion-based systems, particularly since the release pattern may be tailored via the number of β-sheets, degree of crosslinking, or nanoparticle loading [180,181]. SF hydrogels have been shown to be responsive to many stimuli, such as pH, temperature, light, enzymatic cleavage, and redox conditions to permit demand-specific release for use in various biomedical applications [68,182]. Release triggered by NIR has ample precedent in photothermal SF systems in addition to that seen in SF composites containing polydopamine (PDA), although the exact percentage of burst release depends on the composition of the material and protocol of illumination [183]. In addition, magnetic SF hydrogels reinforced with magnetic nanoparticles may allow for remotely triggered release by means of magnetic fields, thus being useful as implantable depots where repeated non-invasive dosing would prove beneficial [23]. The open problem of independent multibiotic release from the same SF platform is yet to be solved.

### 6.5. Wearable and Implantable Bioelectronics

The use of SF hydrogels in flexible electronics is a fast-growing area of application, motivated by the requirement to develop biocompatible, flexible, and potentially self-powered sensing devices [158,184]. Conductive SF hydrogels are particularly promising due to their ion-conducting nature, softness, wet-tissue adhesiveness, biodegradability, and compatibility with wearable or implantable bioelectronics, which makes them different from common metal or silicon-based devices, lacking mechanical matching and biodegradability properties [185,186]. Fu et al. presented an ion-conducting SF-based composite hydrogel that can be used in implantable bioelectronic devices for strain sensing, exhibiting high adhesion, stretchability, and low cytotoxicity in vitro [185]. In the context of pressure sensing, the recently described SF–lignin nanoparticle hydrogel exhibited high sensitivity and fast response, allowing the detection of human movement and pulses [187]. More generally, SF-based conductive hydrogel sensors can be employed in flexible electronics for the development of wearable strain sensors, pressure sensors, and epidermal sensors [158]. One open question is how to integrate different functions in one SF wearable platform, since a fully integrated sensing–actuating drug delivery system is still to come [188].

## 7. Challenges and Future Perspectives

**Figure 10:**
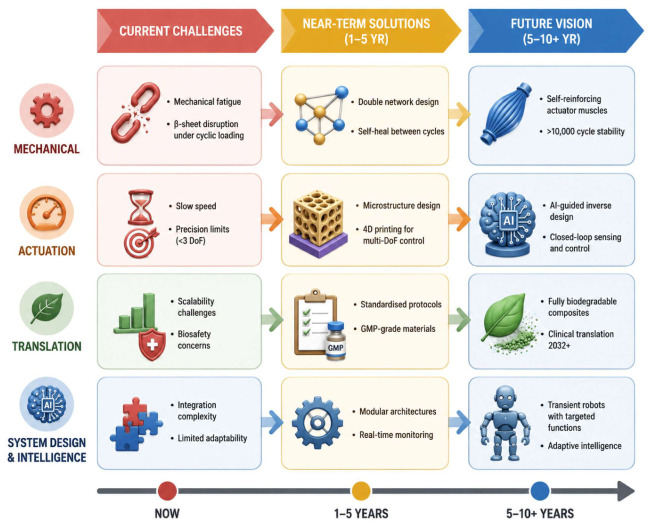
Challenges and future directions roadmap for dynamic SF hydrogel actuators, categorized into three broad areas of themes (mechanical robustness, control of actuation, and clinical application) as well as three different temporal stages. Red-colored boxes are for the quantifiable challenges currently faced, amber-colored boxes for the near-term engineering approaches (within 1 to 5 years), and blue and teal boxes for the futuristic ideas within 5 to 10+ years.

Although much progress has been made in recent times, there are some challenges facing dynamic SF hydrogels that prevent their application in biodevices and soft robots. These challenges include mechanical properties, actuator performance, and translation and clinical applications (Figure 10). Solving these problems will require coordinated advancements in material design, fabrication, and smart systems engineering.

### 7.1. Mechanical Performance

The mechanical robustness of SF hydrogel actuators is still among the most limiting factors. The physical SF hydrogel networks demonstrate the degradation of their storage modulus (G′) from 30% to 50% following 500–1000 loading cycles, caused mostly by the breakage of β-sheet structures and irreversible network reorganization. This fatigue behavior is not sufficient for use in practical applications such as artificial muscles and implantable actuators, where repetitive cycling is necessary. In the short run, the implementation of double-network (DN) hydrogels, where the rigid SF network is embedded within the ductile polymer network (for example, PVA or PAM) network, alongside dynamic covalent crosslinking, will provide an increase in toughness, dissipated energy, and self-healing properties. In the long run, the artificial intelligence-guided design of hydrogel network topology may lead to the creation of self-reinforced actuator muscles that would have the capability of sustaining their performance through more than 10,000 actuation cycles.

### 7.2. Actuation Performance

Currently, SF hydrogel actuators are constrained by low response rates, diffusion-dependent actuation dynamics, and limited multi-degree-of-freedom actuation. Typically, bulk hydrogel-based actuators take several minutes for full actuation, whereas almost all described actuator platforms are confined to simple deformations like bending and contraction. In addition to that, incorporating multiple stimuli within a single device is difficult since multiple stimuli can give rise to an overlap of their response. In the future, microstructure design and 4D printing technology could be used as possible approaches to make SF hydrogel actuators perform faster and allow for a programmable deformation. Also, combining inverse design based on artificial intelligence and self-sensing and feedback systems may be an excellent approach to developing intelligent SF hydrogel actuators with adaptive multi-degree-of-freedom (DoF) actuation.

### 7.3. Translation and Clinical Application

Aside from the lab-based performance metrics, realizing functional devices based on SF hydrogels entails the solution of problems connected with issues of scalability, standardization, biosafety, and regulatory approval of the products. The batch-to-batch inconsistency in the molecular weight, β-sheet fraction, and gel formation of SF, as well as the biocompatibility and degradation of various functional nanomaterials, such as MXene, CNTs, or gold nanoparticles, can serve as examples. Short-term goals include establishing a standardized GMP-compliant manufacturing process, implementing automation, using biodegradable fillers, conducting biocompatibility evaluation, and engaging in regulatory processes to ensure translational capabilities of the technology. In the long run, further progress in biodegradable conductive polymers and fully biodegradable SF composites can result in developing transient soft robots that will completely degrade after performing their therapeutic mission and without the need for surgery. Such development along with AI-based design and scalable manufacturing is likely to expedite the move of SF hydrogel actuators from the laboratory to the clinic.

## 8. Conclusions

Responsive silk fibroin hydrogels have progressed beyond being mere stimulus-responsive biomaterials to become an advanced class of programmable soft matter able to accomplish intricate and designed mechanical functions within biological contexts. The unique set of properties offered by SF material, including reversible β-sheet switching, mechanical tunability, multi-stimuli responsiveness, biocompatibility with FDA approval, and controlled degradability, makes it an exceptional platform of soft matter that cannot be matched by other biomaterials individually. The present review critically analyzes recent developments in six interrelated fields, including molecular structure and physicochemical principles, fabrication and crosslinking methodologies, stimuli-responsive deformation processes, programmable shape-morphing systems, biomedical applications, and translation problems.

A number of important conclusions can be drawn from the critical review. First, the ability of SF to undergo a controllable transition from the random coil to the β-sheet form is not only an advantageous feature but also the underlying mechanism that sets SF apart from all other biomaterials. Unlike GelMA, alginate, or chitosan, SF is a built-in molecular switch capable of responding to at least six different stimuli, thus making it possible to design self-powered actuation systems capable of responding to physiological stimuli without additional electronics. Second, photothermal SF nanocomposites and, specifically, MXene/SF bilayer actuators are the most efficient SF-based actuators reaching a maximum bending angle of 90° in 30 s under NIR irradiation but raising biosafety concerns about the degradation products of MXene. Third, 4D bioprinting using SilMA hydrogels has reached an important breakthrough by being functionalized in vivo, although it remains confined to simple folding actions; for the creation of more complex, multi-step, multi-degree-of-freedom programmed movements, improvements in computation and multi-material printing precision are required. Finally, the applications that will be ready for clinical translation in the near future, smart wound dressings and stimulus-responsive drug delivery systems, are the ones that are already the most advanced in terms of development.

The identified critical issues like fatigue under cyclic stress, diffusion limitation leading to slow response, lack of programmability beyond single mode deformation, signal crosstalk in multi-stimulus systems, variation during the SF fabrication process and incomplete in vivo biosafety characterization of nano-filled systems are interlinked and need to be solved together. To solve these issues, there is a need to take into account the conflicting nature of the performance parameters rather than optimize one parameter. For instance, the use of the double-network structure can be useful for enhancing fatigue resistance and mechanical durability, but may increase the network complexity and delay the diffusion of the stimulus. Similarly, the presence of pores may increase the speed of the response due to the increased mass transfer; however, it may make the material weaker and reduce the actuation force. Hence, there is a need for development using a system-based approach taking into account mechanical toughness, response speed, actuation force, and biological functions.

In terms of future directions, several breakthroughs are on the horizon: the design of SF hydrogels through AI-powered inverse design of desired actuation trajectories; biohybrid structures involving integration of live cells into SF-based matrices to mimic the function of muscle; fully biodegradable transient robots that consist only of biodegradable functional elements used to carry out a specific medical task; and closed-loop self-sensing actuator systems capable of performing self-regulation tasks in a dynamic biological environment. In order to achieve these goals, continued research in an interdisciplinary setting combining materials science, mechanical engineering, computational design, cell biology, and medicine is needed. The unique versatility and the blend of biological and mechanical properties make SF proteins a potential candidate as a material basis for future intelligent biomaterials, even though there are still some improvements needed regarding scalable production and biosafety assessment over the long term and programming the functions of these materials.

## Figures and Tables

**Table 1:** Comparison between SF and other biomaterials as competitors in designing smart hydrogel actuators. GelMA: Gelatin Methacryloyl; PNIPAM: Poly(N-isopropyl acrylamide).

Property	Silk Fibroin	GelMA	Alginate	PNIPAM-Based Hydrogel
Mechanical strength	Tunable 0.1–500 kPa; toughened by β-sheet nanocrystals [46]	10–100 kPa; brittle at high crosslinking	Weak (1–50 kPa) unless ionically reinforced	Moderate (10–100 kPa); decreases above LCST
Biocompatibility	Excellent; FDA-approved; minimal immunogenicity [47,48]	Good; methacryloyl groups may cause mild cytotoxicity	Excellent; widely clinically used	Moderate; NIPAM monomer residues can be toxic
Biodegradability	Tunable days–months via crystallinity control [14]	Enzymatically degradable (MMPs); weeks–months	Rapid unless crosslinked; dissolves in PBS	Non-biodegradable in most formulations
Stimuli responsiveness	Multi-stimuli (pH, T, light, E, B); inherent β-sheet switching [32]	Primarily photo- and enzymatic-responsive; limited dynamic response	pH and ionic; limited temperature response	Excellent thermo-response (LCST ~32 °C); limited multi-stimuli
Actuation performance	Bending angle up to 180°; response time of seconds (photothermal) [49]	Moderate; requires hybrid systems for actuation	Limited; used mainly as passive scaffold	Fast thermal actuation; poor shape memory
Scalability/processing	Requires degumming/dissolution; batch variability [10]	Commercial availability; reproducible	Commercially scalable; low cost	Scalable monomer synthesis; limited biocompatibility
Key advantage for actuation	Inherent conformational switching; versatile nanocomposite integration	High cell adhesion; easy 3D bioprinting	Low cost; clinical track record	Fast, reversible LCST response

**Table 3:** Comparison of representative silk fibroin-based actuators. (NR: Not reported in the accessible literature).

SF-Based System	Stimulus	Actuation Mechanism	Deformation Mode	Actuation Performance	Response Time	Cycle Stability	Application	Ref.
Regenerated silk hierarchical actuator	Humidity gradient	Water adsorption/desorption-induced molecular rearrangement and hierarchical amplification	Flipping locomotion, self-oscillation, 2D–3D transformation	Water-responsive shape recovery rate is about 83%; maximum actuation stress reaches up to 18 MPa; strong toughness	Cyclic humidity response, exact switching time = NR	Reversible cyclic response reported	Soft actuators, artificial muscles	[155]
SF shape memory composite hydrogel	Environmental programming/recovery stimulus	SF-regulated semi-IPN network and reversible shape memory transition	Shape fixation and recovery	Tunable recovery time depending on SF content; improved network stability	Tunable recovery kinetics	Reported as durable and controllable	Soft grippers, adaptive devices	[134]
SF–PNIPAM composite hydrogel	Temperature	LCST-driven polymer collapse reinforced by SF network	Volume contraction	Thermo-responsive collapse; SF increases stability and modulates deswelling kinetics	Minutes scale, composition-dependent	Improved deswelling kinetics and stability versus PNIPAM alone	Thermal actuators, drug delivery	[156,157]
SF–MXene hydrogel	NIR/electrical stimulus	Photothermal conversion and conductive-network response	Bending, contraction	Quantitative values vary by architecture; used for fast soft actuation	Seconds to minutes, architecture-dependent	Reversible actuation reported in related conductive SF systems	Soft robotics, sensors	[85,158]
SF–GO composite hydrogel	Light/thermal stimulus	Photothermal conversion plus mechanical reinforcement	Bending, folding	Improved mechanical robustness and actuation reliability compared with SF alone	Seconds to minutes	Better durability than SF alone	Artificial muscles	[83,159]
SF–Au nanoparticle hydrogel	NIR irradiation	Localized photothermal heating	Contraction, bending	NIR-driven actuation; quantitative values not fixed across designs	Seconds to minutes	NR	Biomedical actuation and therapy	[160,161]
SF–Fe_3_O_4_ magnetic hydrogel	Magnetic field	Magnetic torque and particle alignment	Directional deformation, motion	Magnetic actuation is directional and rapid; exact force depends on loading	Seconds	Reversible magnetic actuation reported	Soft magnetic robots	[123,162]
SF double-network hydrogel	Mechanical stimulation	Interpenetrating network reinforcement	Large deformation, recovery	Enhanced toughness and force output; performance depends on network composition	Slower than single networks	High cyclic durability	Artificial muscles	[104,163]
4D-printed SF hydrogel systems	Programmed external stimulus	Spatially controlled architecture and anisotropic deformation	Folding, twisting, shape transformation	Architecture-dependent	Architecture-dependent	Depends on formulation	Biomedical devices and soft robotics	[164,165]

## Data Availability

No new data were created or analyzed in this study.
